# The Influence of Apremilast-Induced Macrophage Polarization on Intestinal Wound Healing

**DOI:** 10.3390/jcm12103359

**Published:** 2023-05-09

**Authors:** Annika Mohr, Manuela Besser, Sonja Broichhausen, Maximiliane Winter, Alexander D. Bungert, Benjamin Strücker, Mazen A. Juratli, Andreas Pascher, Felix Becker

**Affiliations:** Department of General, Visceral and Transplant Surgery, University Hospital Münster, 48149 Münster, Germany

**Keywords:** apremilast, IBD, intestinal wound healing, macrophages, monocytes, NF-κB, THP1

## Abstract

There is compelling evidence suggesting a pivotal role played by macrophages in orchestrating intestinal wound healing. Since macrophages display significant plasticity and heterogeneity, exhibiting an either classically activated (M1-like) or alternatively activated (M2-like) phenotype, they can aggravate or attenuate intestinal wound healing. Growing evidence also demonstrates a causal link between impaired mucosal healing in inflammatory bowel disease (IBD) and defects in the polarization of pro-resolving macrophages. By targeting the switch from M1 to M2 macrophages, the phosphodiesterase-4 inhibitor Apremilast has gained recent attention as a potential IBD drug. However, there is a gap in our current knowledge regarding the impact of Apremilast-induced macrophages’ polarization on intestinal wound healing. The THP-1 cells were differentiated and polarized into M1 and M2 macrophages, and subsequently treated with Apremilast. Gene expression analysis was performed to characterize macrophage M1 and M2 phenotypes, and to identify possible target genes of Apremilast and the involved pathways. Next, intestinal fibroblast (CCD-18) and epithelial (CaCo-2) cell lines were scratch-wounded and exposed to a conditioned medium of Apremilast-treated macrophages. Apremilast had a clear effect on macrophage polarization, inducing an M1 to M2 phenotype switch, which was associated with NF-κB signaling. In addition, the wound-healing assays revealed an indirect influence of Apremilast on fibroblast migration. Our results support the hypothesis of Apremilast acting through the NF-κB-pathway and provide new insights into the interaction with fibroblast during intestinal wound healing.

## 1. Introduction

The intestinal mucosa serves as a selective barrier separating foreign contents from the internal host milieu and the integrity of this barrier is a pivotal prerequisite for intestinal homeostasis [1]. Upon injury, mucosal wound healing begins with an initial inflammatory response followed by regeneration and repair, aiming to promote the rapid re-epithelialization, elicited by the adhesion, migration, proliferation, and differentiation of intestinal epithelial cells (IEC) at the wounded area [2]. This tightly regulated process depends on a precisely orchestrated series of events that integrate not only IEC but also mesenchymal cells, especially lamina propria fibroblasts, which shift from inflammation-related, potentially fibrotic, activity to the synthesis of proteins and proteoglycans as extracellular matrix (ECM) components to re-establish the intestinal structure [3]. Macrophages are critical players in coordinating cellular processes upon injury [4]. In response to the signals received from their immediate environment, resident or infiltrated macrophages undergo dramatic phenotypic and functional changes [5] that are simplified by the in vitro M1-like (classically activated) and M2-like (alternatively activated) model [6,7]. Thus, they contribute to inflammation (M1-like) but also initiate and maintain tissue remodeling and regeneration (M2-like) [8,9]. Further, they can be key modulators in cancer progression, contributing to tumor-related inflammation (M1- and M2-like) and the promotion of angiogenesis, neovascularization, stromal activation, and remodeling (M2-like) [10,11,12,13,14]. 

The polarization of macrophages in vitro toward an M1-like type is induced by pathogen-associated molecule patterns, such as bacterial lipopolysaccharides, and by inflammation-related cytokines TNF-alpha or IFN-gamma. In contrast, M2-like polarization is induced in response to the Th2-related cytokines IL-4 or IL-13 [15,16]. 

There is now compelling evidence suggesting a pivotal role played by classically activated and alternatively activated macrophages in governing intestinal wound healing [17,18]. The M1-like macrophages are effective producers of toxic effector molecules (ROS and NO) and inflammatory cytokines (IL1β, TNF, IL6), thus presenting a proinflammatory phenotype [15,16]. They aggravate epithelial damage and are responsible for the phagocytosis of cellular debris from the wound environment, followed by dampening inflammatory signals and initiating the proliferative phase of mucosal repair [6,19]. In contrast, M2-like macrophages represent a phenotype that is potentially important in intestinal wound healing by tissue remodeling and resolving inflammation [16]. Through the secretion of anti-inflammatory mediators (especially IL10) and growth factors (PDGF, TGF-β), they promote IEC renewal and aid in tissue healing via stabilizing angiogenesis and stimulating progenitor cell proliferation [20]. Through their ability to secrete cytokines and growth factors such as matrix metalloproteinases (MMPs) or transforming growth factors (TGFBs), they stimulate fibroblasts to proliferate, differentiate, and migrate to constitute a new ECM [21,22,23]. These observations and the emerging evidence imply that M2-like macrophages can mediate mucosal healing by releasing mediators to orchestrate the proliferation and migration of wound-associated cells, including fibroblasts.

Recent findings suggest a causal link between inflammatory bowel diseases (IBD) and a failure in the resolution of inflammation, evident in defects of the transition from M1-like to M2-like macrophages, resulting in sustained inflammation and impaired mucosal healing [24]. Considering their unique ability to undergo phenotypic and functional changes during wound healing, macrophages have gained recent attention as therapeutic targets in IBD to enhance intestinal wound healing [6]. The novel and potential future IBD drug Apremilast (a phosphodiesterase-4 (PDE4) inhibitor) has been shown to target a macrophage phenotype switch (M1 to M2) by down-regulating inflammatory responses through the inhibition of NF-κB transcriptional activity and NF-κB -dependent genes [23,24,25], and has already been successfully tested in a phase II trial in patients with active ulcerative colitis (UC) [26]. However, the influence of Apremilast-induced changes on macrophage polarization, the underlying genetic mechanisms, and their impact on intestinal wound healing have not been investigated yet.

## 2. Materials and Methods

### 2.1. Cell Culture

THP-1 (human leukemic monocytes) cells were maintained in culture in Eagle’s Minimum Essential Medium (EMEM, ROTI^®^Cell, Carl Roth, Karlsruhe, Germany) containing 10% of heat-inactivated fetal bovine serum (FBS, Sigma-Aldrich, Taufkirchen, Germany), supplemented with L-Glutamine (20 nM, L-Glutamine solution, Sigma-Aldrich) and ß-mercaptoethanol (0.5 mM, Sigma-Aldrich). A standardized protocol, previously published by Genin et al., was used for the differentiation and polarization of THP-1 monocytes into macrophages [27]. Cells (10 × 10^5^) were seeded to 6-well plates and their differentiation into M0 macrophages was initiated via a 24 h incubation (37 °C, 5% CO_2_) with phorbol 12-myristate 13-acetate (PMA, 500 ng/mL, Sigma-Aldrich). Macrophages were polarized into M1 macrophages by incubation with PMA (10 ng/mL), lipopolysaccharides (LPS, 100 ng/mL, from *Escherichia coli* O111:B4, Sigma-Aldrich), and interferon-gamma (IFN-γ, 20 ng/mL, Sigma-Aldrich), or into M2 macrophages by incubation with PMA (10 ng/mL), IL-4 (20 ng/mL, Sigma-Aldrich), and IL-13 (20 ng/mL, Sigma-Aldrich) for 24 or 48 h, respectively. CaCo-2 (immortalized human colorectal adenocarcinoma) and CCD-18 (human colonic fibroblasts, CCD18Co (ATCC^®^ CRL1459), ATCC™) cells were maintained in culture in EMEM (Carl Roth) containing 10% of heat inactivated FBS (Sigma-Aldrich), supplemented with L-Glutamin (20 nM, Sigma-Aldrich).

### 2.2. Histology

THP-1 cells were seeded to transwells in 6-well plates. After reaching confluency, cells were stained with Gill’s Hematoxylin Solution and observed under a microscope (brightfield, Keyence BZ-X800, Keyence Deutschland GmbH, Neu-Isenburg, Germany).

### 2.3. Immunofluorescence Labeling

THP-1 cells (10 × 10^5^) were seeded to 6-well plates. Cells were then washed with phosphate buffered saline (PBS, PAN-Biotech GmbH, Aidenbach, Germany) and incubated with 4 °C-cold paraformaldehyde-solution (4% PFA, Morphisto GmbH, Offenbach am Rhein, Germany) for five minutes at room temperature. After washing with PBS, cells were permeabilized with PBT 1 (PBS, 0,2% Triton X, 10 g/L bovine serum albumin (BSA)) for at least 20 min. Primary antibodies (CD71 (mouse, Santa Cruz Biotechnology, Dallas, USA) and CD68 (mouse Santa Cruz Biotechnology,)) were incubated for 30 min following washing with PBS/A (PBS, 1 g/L BSA) and incubation with the secondary antibody (mouse IgG κ binding protein conjugated with CruzFluor™ 555, Santa Cruz Biotechnology). Cells were counterstained with DAPI (Thermo Scientific, Schwerte, Germany, 10 ng/mL), incubated for another 30 min and washed twice with PBS. The samples were then mounted in an Immu-Mount (Epredia, Microm International GmbH, Dreieich, Germany), fixed with coverslips and observed under a fluorescence microscope (Keyence BZ-X800, Keyence).

### 2.4. Apremilast Treatment

For this, 10 mg of Apremilast (Otezla^®^, Amgen, Germany) was dissolved in 10 mL dimethyl sulfoxide (DMSO) and sterile filtrated. For treatment, THP-1 cells were seeded to 6-well plates and polarization was conducted as described above. Next, 4.6 µL of Apremilast solution was added to 10 mL of EMEM (Carl Roth) to obtain a concentration of 1 µmol/L and was added to the cells for 90 min. After washing with PBS, cells were again incubated for 24 h with a normal culture medium. For the control group, cells were kept in a normal culture medium without treatment. Cell pellets of untreated and treated M1 and M2 macrophages were subsequently frozen at −80 °C for further analysis. Furthermore, the conditioned medium of cells with or without Apremilast treatment was collected, sterile filtrated (0.45 µm and 0.22 µm Filter, Millex Filter with MCE Membrane, Merck Millipore, Molsheim, Germany), and frozen at −20 °C for further analysis.

### 2.5. Real-Time Quantitative PCR

Complete RNA was extracted from frozen cell pellets using the AllPrep RNA/Protein Kit (Qiagen, Hilden, Germany). The First Strand cDNA Synthesis Kit (ThermoScientific) was then used for cDNA synthesis according to the manufacturer’s protocol. The dilution for real-time qPCR was prepared by adding SsoAdvanced Universal SYBR Green Supermix (Bio-rad, Feldkirchen, Germany) and primers (Metabion interational AG, Planegg/Steinkirchen, Germany): CD206 (for 5′-TTC GGA CAC CCA TCG GAA TTT-3′, rev 5′-CAC AAG CGC TGC GTG GAT-3′), tumor necrosis factor alpha (TNFα) (for 5′-AGC CCA TGT TGT AGC AAA CC-3′, rev 5′-TAG ATG GGC TCA TAC CAG GG-3′), CCL22 (for 5′-ATT ACG TCC GTT ACC GTC TG-3′, rev 5′-TAG GCT CTT CAT TGG CTC AG-3′), IL10 (for 5′-TAC GGC GCT GTC ATC GAT TT-3′, rev 5′-TAG AGT CGC CAC CCT GAT GT-3′), IL1β (for 5′-GGA CAA GCT GAG GAA GAT GC-3′, rev 5′ TCT TTC AAC ACG CAG GAC AG-3′), IL6 (for G’GAC AGC CAC TCA CCT CTT CA-3′, rev 5′-AGT GCC TCT TTG CTG CTT TC-3′), and GAPDH (for 5′-CCC TCC AAA ATC AAG TGG-3′, rev 5′-CCA TCC ACA GTC TTC TGG-3′). The qPCR was realized by CFF96 Touch Real-Time System (Bio-rad). The expression rates were normalized to the housekeeping gene GAPDH and were evaluated by applying the 2^−ΔΔCt^ method.

### 2.6. Next Generation Sequencing (NGS)

Total RNA was isolated from 3 cell pellets of each group (M1 and M2 with and without Apremilast treatment) using the AllPrep RNA/Protein Kit (Qiagen), according to the manufacturer’s instructions and following an additional purification step. The RNA purity was then ensured by determining the absorbance ratio at 260 and 280. The library preparation of the total RNA was performed with the NEBNext Ultra II RNA directional Kit and single-read sequencing was performed using a NextSeq^®^ 2000 System with a read length of 72 bp. Using a molecular barcode, the samples were demultiplexed (bcl convert 3.8.4) to fastq data and quality controlled (FastQC). Trimmomatic was used for adapter trimming and read filtering [28]. The resulting reads were aligned to the reference genome using Hisat2 [29]. The aligned reads were sorted using samtools [30]. The sorted and aligned reads were counted into genes using htseq-counts [31]. The test for differential expression was performed using the r-package deseq2 [32]. Services were performed at the Core Facility Genomics of the Medical Faculty, Westfälische Wilhelms-Universität Münster.

RNA libraries were screened for the target genes of macrophage polarization and fibroblast-macrophage interactions based on the literature [23,33,34,35]. Data were presented as either heatmaps or column diagrams. Then, to understand the biological processes and the pathways involved, GO (Gene Ontology) enrichment analysis was performed on the DAVID database [36].

### 2.7. Wound Healing Assay

To determine the influences of M1 and M2 macrophages with or without Apremilast treatment on fibroblasts or IEC migration, scratch assays were performed to mimic intestinal wound healing in vitro. For scratch assays, 24-well plates were coated with collagen (Type I, Sigma-Aldrich), incubated for two hours at 37 °C, and washed with PBS. Then, CCD18 or CaCo2 cells (100,000) were seeded to the wells and incubated overnight. After reaching a confluent monolayer, Mitomycin C (PanReac. AppliChem, 10 µg/mL) was added and incubated for another two hours. Scratches were conducted using a 200 µL pipet tip and the cells were washed twice with PBS to remove the detached cells. Wells were then filled with 800 µL culture medium and 200 µL conditioned medium. The wells filled with 100% culture medium served as controls. The first pictures were obtained immediately using phase contrast (Keyence BZ-X800) for two different areas and for every 6 h in 48 h. Experiments were performed in triplets.

### 2.8. Statistical Analysis

For statistical analysis, unpaired two-tailored *t*-tests were performed comparing two groups, considering *p* < 0.05 as statistically significant and presenting data as mean ± SEM. A statistical analysis for wound healing assays was performed by a one-way ANOVA with a Tukey multiple for each time point. The differences were considered statistically significant at a value of *p* < 0.05. Graphs were depicted with GraphPad Prism 9 (GraphPad Software, San Diego, CA, USA).

## 3. Results

### 3.1. Monocyte-to-Macrophage Differentiation

**Figure 1:**
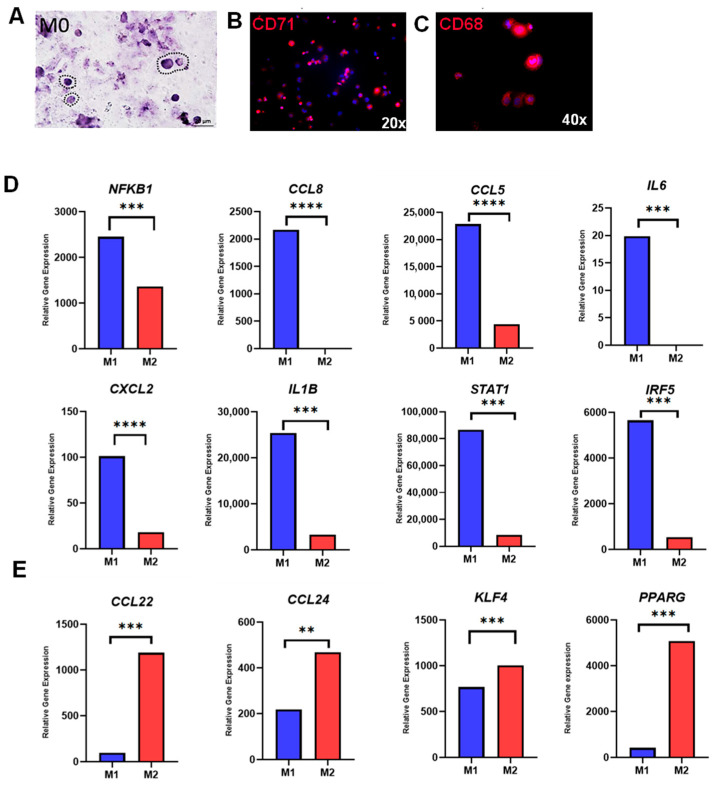
Monocyte-to-macrophage differentiation and polarization. (**A**) Representative M0 macrophages with HE-staining (40× magnification); (**B**) Immunofluorescence staining of M0 macrophages with CD71 (20× magnification); (**C**) Immunofluorescence staining of M0 macrophages with CD68 (40× magnification); (**D**) Relative gene expression of M1-associated genes (*NFKB1*, *CCL8*, *CCL5*, *IL6*, *CXCL2*, *IL1B*, *STAT1*, and *IRF5*); and (**E**) Relative gene expression of M2-associated genes (*CCL22*, *CCL24*, *KLF4*, *PPARG*). * *p* < 0.05, ** *p*  <  0.01, *** *p*  <  0.001 and **** *p* < 0.0001.

First, it was tested whether human THP-1 monocytes could be differentiated from M0 macrophages and subsequently further polarized to M1 and M2 macrophages. Following PMA incubation, monocyte-to-macrophage differentiation was initially tested using histology and immunofluorescence. The THP-1 cells became adherent and showed typical macrophage morphology (Figure 1A). The expression of established macrophage markers CD71 (Figure 1B) and CD68 (Figure 1C) confirmed the successful monocyte-to-macrophage differentiation.

### 3.2. M1 and M2 Macrophage Polarization

Next, the polarization from M0 to M1 or M2 macrophages was examined using the established protocol by Genin et al. [27]. Following polarization, RNAseq data from M1 and M2 macrophages were screened for well-established M1- and M2-specific genes, revealing a distinct expression pattern for each group. Genes that were highly associated with M1 polarization (*IL1β*, *IL6*, *NFKB1*, *CCL8*, *CCL5*, *CXCL2*, *STAT1*, and *IRF5*) were significantly upregulated in M1 macrophages treated with LPS and IFN-γ (Figure 1D). In addition, samples of M2 macrophages treated with IL-4 and IL-13 exhibited significantly higher expression of characteristic M2 genes (*KLF4*, *PPARG*, *CCL22*, and *CCL24*), indicating successful polarization from M0 to M1 and M2 macrophages (Figure 1E).

### 3.3. The Effect of Apremilast on Macrophage Polarization

**Figure 2:**
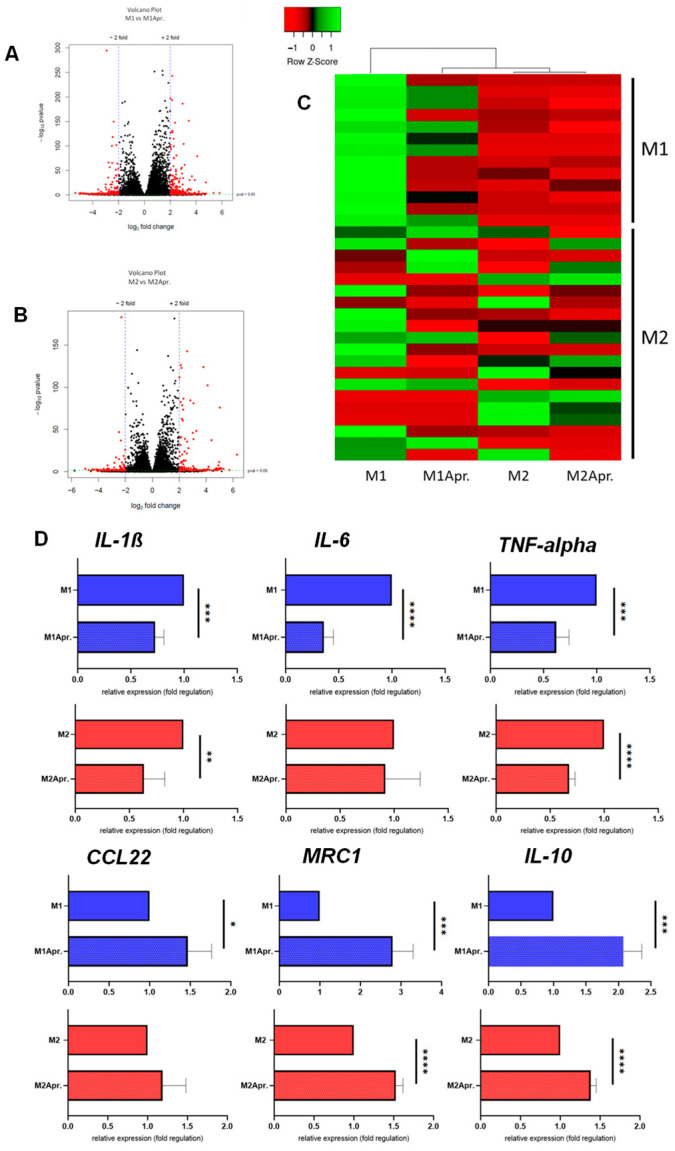
Differences in gene expression due to Apremilast treatment. Volcano Plot of the comparison between (**A**) untreated M1 (M1) vs. Apremilast-treated (M1Apr.) M1 macrophages and (**B**) untreated M2 (M2) vs. Apremilast-treated (M2Apr) M2 macrophages. (**C**) Genes associated with M1 and M2 macrophage polarization were plotted in the heat map. Expression levels are depicted according to the color scale in the top left corner. Row scaling and hierarchical clustering on the Pearson distance was performed. The hierarchical clustering is visualized by the dendrogram at the top, which illustrates the degree of relatedness in gene expression between samples. Complete list of genes shown in Table S1 (**D**) Gene expression analysis of *IL-1β*, *IL-6*, *TNF-alpha*, *CCL22*, *MRC1* and *IL-10* comparing M1 and M2 macrophages with or without Apremilast treatment. Results are shown as relative gene expression (fold regulation). * *p*  <  0.05, ** *p* <  0.01, *** *p*  <  0.001 and **** *p* < 0.0001.

Having established a successful in vitro polarization of M1 and M2 macrophages, it was next thought to test the effect of Apremilast on macrophage polarization. The M1 and M2 macrophages were treated with Apremilast, and subsequently analyzed by RNAseq and selected genes by qPCR. Volcano Plots show differentially expressed genes (DEG) between groups (Figure 2A,B). In more detail, among the 58,396 analyzed genes, 13% (7690) were differentially expressed when comparing M1 macrophages with M1 macrophages treated with Apremilast. Thereof, 56% were upregulated and 44% were downregulated. Further, 17.5% (10,231) of all analyzed genes showed significant differences when comparing M2 macrophages with M2 macrophages treated with Apremilast. Out of these, 5170 (50.5%) DEGs were significantly upregulated and 5061 (49.5%) were significantly downregulated. Hereby, we could demonstrate a clear effect of Apremilast on M1 and M2 macrophage gene expression.

Next, genes known to be involved with M1/M2 polarization were plotted in a heat map. As shown in Figure 2C, a distinct separation between the untreated and Apremilast-treated M1 macrophages was found for M1 associated genes, accompanied by a clear clustering of Apremilast-treated M1 macrophages with the untreated and Apremilast-treated M2 macrophages. These results indicate that Apremilast treatment in M1 macrophages resulted in a change of gene expression profile towards the M2 macrophage gene expression signature. Upon closer analysis of the selected genes (*IL-6*, *TNF-α*, *IL-1β*, *CCL22*, *MRC1* and *IL-10*) (Figure 2D), we found M1 macrophages treated with Apremilast to experience a significant downregulation of genes associated with the M1 genotype (*IL-6*, *TNF-α*, and *IL-1β*). Moreover, M2-associated genes (*MRC1*, *CCL22*, and *IL-10*) were significantly upregulated in both M1 and M2 macrophages after the Apremilast treatment. These results suggest an Apremilast-elicited phenotype switch from M1 to M2 macrophages.

**Figure 3:**
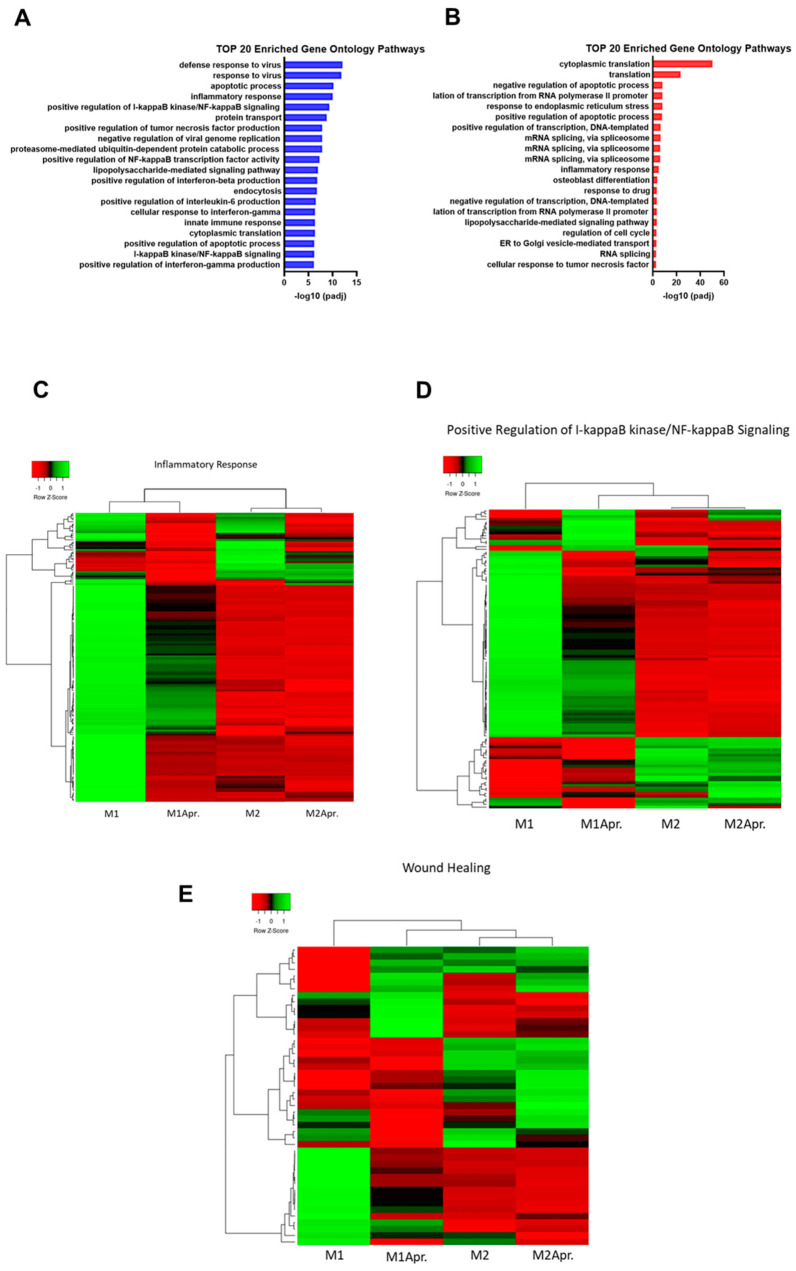
Pathway analysis of M1 and M2 Macrophages with or without Apremilast treatment. (**A**) Top 20 enriched Gene Ontology Pathways (biological process) of upregulated genes comparing untreated M1 with Apremilast-treated (M1Apr) M1 macrophages. (**B**) Top 20 enriched Gene Ontology Pathways (biological process) of upregulated genes comparing untreated M2 with Apremilast-treated (M2Apr) M2 macrophages. (**C**) Differentially expressed genes of GO:0006954 (inflammatory response) were plotted in a heatmap. Expression levels are depicted according to the color scale in the top left corner. Row scaling and hierarchical clustering on the Pearson distance was performed. The hierarchical clustering is visualized by the dendrogram at the top, which illustrates the degree of relatedness in gene expression between samples. Complete list of genes shown in Table S1. (**D**) DEG of GO:0043123 (positive regulation of I-κB kinase/NF-κB signaling) was plotted in a heatmap. Expression levels are depicted according to the color scale in the top left corner. Row scaling and hierarchical clustering on the Pearson distance was performed. The hierarchical clustering is visualized by the dendrogram at the top, which illustrates the degree of relatedness in gene expression between samples. Complete list of genes shown in Table S1. (**E**) DEG of GO:0042060 (wound healing) was plotted in a heatmap. Expression levels are depicted according to the color scale in the top left corner. Row scaling and hierarchical clustering on the Pearson distance was performed. The hierarchical clustering is visualized by the dendrogram at the top, which illustrates the degree of relatedness in gene expression between samples. Complete list of genes shown in Table S1.

To further explore the biological functions of DEGs and supraordinate regulation mechanisms, gene set enrichment analysis was performed and the top 20 most significant GO terms (biological process (BP) category)) of the upregulated genes comparing untreated and Apremilast-treated M1 and M2 macrophages, respectively, were analyzed. For M1 macrophages, we found multiple pathways that were involved in inflammation and macrophage-associated effects were among the top 20 enriched gene ontology pathways (Figure 3A). Inflammatory response (GO:0006954) and NF-κB signaling (GO:0043123, GO:0007249), which has been shown to be crucial for macrophage-associated effects during impaired wound healing, were found among the top 20 pathways. Further relevant enriched pathways were TNF- (GO:0032760), IL6- (GO:0032755), and IF gamma production, all of which are known to be the mediators of proinflammatory responses. Next, DEG of GO:0043123 (positive regulation of I-kappaB kinase/NF-kappaB signaling) and GO:0006954 (inflammatory response) were further analyzed and plotted as a heatmap. An analysis of DEGs within the inflammatory response pathway (GO:0006954) showed a down-regulation for Apremilast-treated M1 macrophages, M2, and Apremilast-treated M2 macrophages, when compared with untreated M1 macrophages (Figure 3C). As shown in Figure 3D, a distinct separation between untreated and Apremilast-treated M1 macrophages was found for NF-κB signaling, accompanied by the clear clustering of Apremilast-treated M1 macrophages with untreated and Apremilast-treated M2 macrophages. These results indicate that Apremilast treatment in M1 macrophages resulted in a downregulation of genes involved in NF-κB signaling, which led to a M2-like gene expression profile (Figure 3D). Given the paramount role played by NF-κB signaling in wound healing, the expression of established wound healing–associated genes (GO:0042060 (wound healing)) was compared between untreated and Apremilast-treated M1 and M2 macrophages. Again, it was found that Apremilast elicited distinct changes in the expression of wound healing–associated genes in M1 macrophages, which clearly clustered with the untreated and Apremilast-treated M2 macrophages (Figure 3E).

### 3.4. Effect of Apremilast-Treated Polarized Macrophages on Intestinal Wound Healing In Vitro

**Figure 4:**
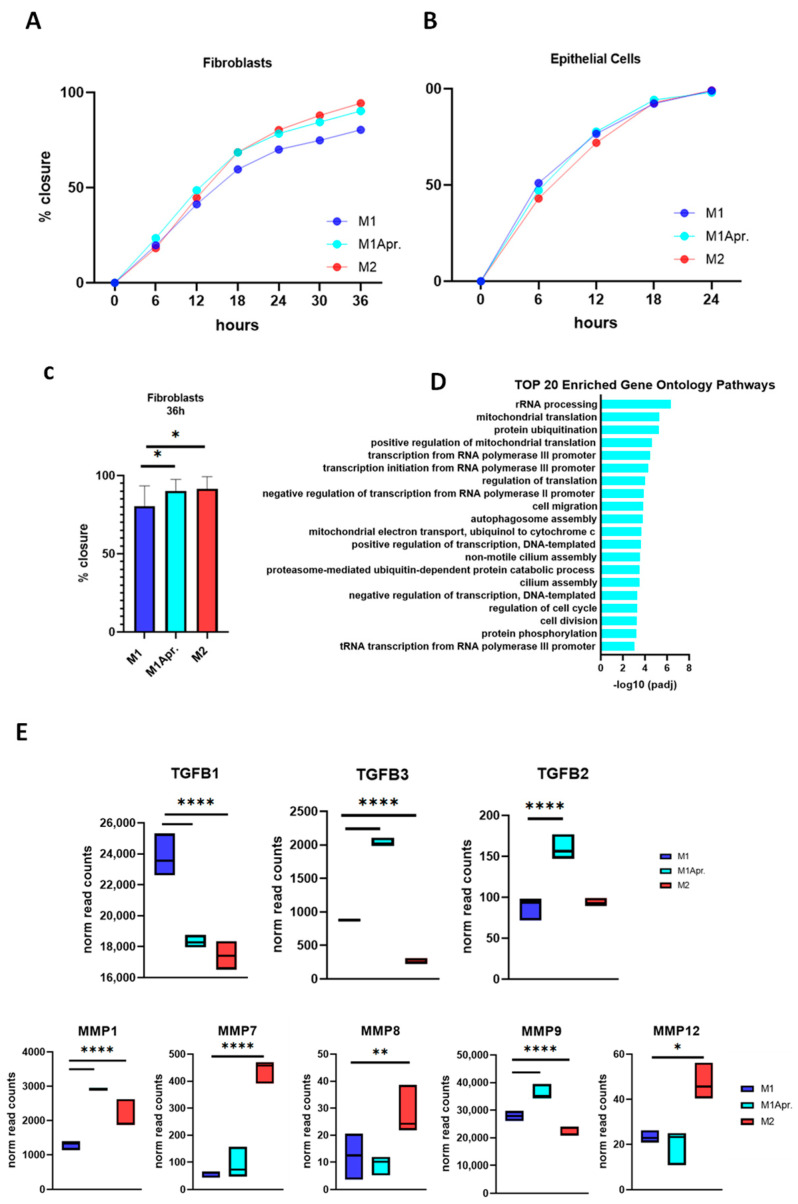
Influence of paracrine factors from untreated M1, Apremilast-treated M1, and untreated M2 on cell migration. (**A**) Wound closure (%) of epithelial cells incubated with conditioned medium of either untreated M1/M2 macrophages or Apremilast-treated (M1 + Apr.) M1 macrophages for 24 h. (**B**) Wound closure (%) of fibroblasts incubated with conditioned medium of either untreated M1/M2 macrophages or Apremilast-treated (M1 + Apr.) M1 macrophages for 36 h. (**C**) Wound closure (%) of fibroblasts at time point 36 h. * *p* <  0.05. (**D**) Top 20 enriched Gene Ontology Pathways (biological process) of upregulated genes (M1Apr macrophages). (**E**) Normalized read count of Transforming Growth Factors (*TGFB1*, *TGFB2*, and *TGFB3*) and Matrix Metalloproteinases (*MMP1*, *MMP7*, *MMP8*, *MMP9*, and *MMP12*) for untreated M1, Apremilast-treated M1 (M1Apr), and untreated M2 macrophages. * *p*  <  0.05, ** *p*  <  0.01 and **** *p* < 0.0001.

It was next thought to test the effect of paracrine factors of Apremilast-treated macrophages on intestinal wound healing in vitro. Fibroblast and IECs were cultured with a conditioned medium of untreated M1 macrophages, Apremilast-treated M1 macrophages, and untreated M2 macrophages. Wound-healing assays were then performed to analyze their influence on cell migration. For IECs, a rather rapid artificial wound closure was observed, independent of the applied conditioned medium. Generally, epithelial cells showed a tendency toward faster cell migration compared with fibroblast cells and tended to close the artificial scratch after about 18 h (Figure 4A,B).

In contrast, fibroblasts’ cell migration was decelerated compared with IECs. Notably, there was a clear influence of untreated M2 macrophages and Apremilast-treated M1 macrophages on fibroblast migration, with the conditioned medium promoting wound closure (Figure 4B). In comparison with the conditioned media derived from untreated M1 macrophages, treatment with conditioned media from Apremilast-treated M1 macrophages and M2 macrophages significantly improved wound closure after 36 h (Figure 4C).

In order to identify the role of Apremilast in macrophage-dependent fibroblast migration, a GO enrichment analysis was performed, focusing on upregulated genes in Apremilast-treated M1 macrophages. Comparing enriched GO terms in the Biological Process (BP) category, we found that cell migration (GO:0016477) and cell division (GO:0051301) were among the top 20 most enriched GO terms pathways (Figure 4D), suggesting that through macrophage activation, Apremilast may mediate the effect on these specific pathways and thus ameliorate fibroblast migration. Given the fundamental role of macrophages in fibroblast activation, proliferation, and migration, it was next thought to analyze the appendant genes of macrophages upon fibroblast activation. Macrophages are leading producers of matrix MMPs and TGFs, both known to act as important signals in macrophage–fibroblast interaction. When analyzing the gene expression of MMPs (MMP1, MMP7, MMP8, MMP9, and MMP12) and TGFBs (TGFB1, TGFB2, and TGFB3), we found MMP9 and TGFB2 among the DEG of the cell migration pathway, showing their critical involvement in wound closure in the present study. Moreover, by single analysis, both MMP9 and TGFB2 were significantly upregulated in Apremilast-treated M1 macrophages compared with the control. Similar results were found for MMP1 and TGFB3, suggesting a key influence of those genes on fibroblast cell migration within in vitro wound closure.

## 4. Discussion

The gastrointestinal mucosa serves as a selective barrier separating foreign contents from the internal host milieu [1]. Upon injury, intestinal wound healing aims to promote mucosal re-epithelialization. This process is regulated by the local, mechanical and chemical crosstalk between IEC and laminar propria fibroblasts [2]. Additionally, there is compelling evidence suggesting a crucial role of macrophages in orchestrating intestinal wound healing [18]. Thereby, macrophages display a significant plasticity and heterogeneity, exhibiting either a classically activated (M1-like) or alternatively activated (M2-like) phenotype [5]. In the present study, we focused on the influence of the PDE4 inhibitor Apremilast on the polarization of M1 and M2 macrophages and demonstrated the clear impact of Apremilast treatment on M1 and M2 gene expression signatures. This effect was associated with a phenotype switch from M1 to M2 macrophages after Apremilast treatment and the downregulation of genes involved with NF-κB–signaling and inflammatory response. Moreover, Apremilast-treated macrophages improved fibroblast migration in an in vitro wound healing model.

### 4.1. Macrophage Switch by Apremilast Treatment

Our results indicate that Apremilast treatment can target the phenotype switch from M1 to M2 macrophages. Through the inhibition of PDE4, Apremilast elevates intracellular cAMP levels, resulting in the down-regulation of inflammatory responses by reducing the expression of TNF-α, IL-23, and other pro-inflammatory cytokines. Collaterally, anti-inflammatory cytokines such as IL-10 are increased [37,38]. Thus, the inhibition of PDE4 represents an interesting therapeutic approach for inflammatory conditions. Apremilast has been approved for the treatment of patients with psoriatic arthritis, moderate to severe plaque psoriasis and oral ulcers of Behçet disease [39,40,41]. Its potential in the treatment of inflammatory and autoimmune cutaneous dermatoses is demonstrated by its off-label usage in treating various dermatological inflammatory diseases, including atopic dermatitis, chronic cutaneous sarcoidosis, lichen planus, and chronic aphthous stomatosis [41,42,43]. In blood samples from patients with psoriasis, Apremilast reduced the production of pro-inflammatory cytokines and increased the levels of anti-inflammatory mediators [44]. Another study examining Apremilast in inflammatory conditions clearly demonstrated the effect of Apremilast on restraining the polarization of M1 macrophages, and thus, decreasing the infiltration of inflammatory monocytes in skin fibrosis [37]. Given the promising results obtained in dermatology, Apremilast has already been investigated in intestinal healing processes: in intestinal inflammation, namely UC, Apremilast was found to be protective by interfering with mucosal immunity [25]. Furthermore, Apremilast suppressed LPS-stimulated inflammatory responses in macrophages. In the present study, we could demonstrate a phenotype switch from M1 to M2 macrophages that was targeted by Apremilast. This is of paramount importance in IBD, since an M1/M2 imbalance has been identified to be crucially involved in its pathogenesis [24]. Therefore, therapeutic approaches that target the imbalance of macrophage plasticity would be essential for appropriate intestinal wound healing. Accordingly, Apremilast has been successfully tested in a phase II trial in patients with active UC in which mucosal healing was among the endpoints [26].

### 4.2. The Effect of Apremilast on NF-κB Signaling Pathway in Macrophages

In addition, our data also revealed a mechanism of action for Apremilast by demonstrating not only its involvement in NF-κB signaling but also in inflammatory response. The downregulation of pathway-associated genes demonstrates the Apremilast-elicited anti-inflammatory effect on macrophages. This effect of Apremilast is based on the inhibition of PDE4. This enzyme predominantly hydrolyzes cAMP in mammalian cells. The increase of intracellular cAMP through Apremilast leads to phosphorylation and the activation of PKA, resulting in the up-regulation of CREB and inhibition of NF-κB transcriptional activity and NF-κB-dependent genes [38]. In the present study, Apremilast clearly had an impact on M1 macrophages and the NF-κB–signaling pathway. NF-κB is an important signaling cascade that drives M1 activation in macrophages [45]. After stimulation, NF-κB transcription factors are translocated into the nucleus following downstream effector mechanisms and the induction of proinflammatory mediators, such as the TNF-alpha and IL-6—genes that were significantly down-regulated in M1 macrophages after Apremilast treatment in our study. Accordingly, GO term enrichment analysis showed that Apremilast treatment affects the inflammatory response by downregulating the related genes in Apremilast-treated M1 macrophages.

### 4.3. Positive Effects on Wound Healing In Vitro and Fibroblast Migration Mediated by Apremilast-Treated Polarized Macrophages

Since 2014, Apremilast has been approved for the treatment of psoriatic arthritis and plaque psoriasis due to its beneficial results in the reduction of clinical signs of inflammation. Ongoing studies continue to evaluate the potential of Apremilast on wound healing and inflammatory processes. When analyzing the impact of polarized macrophages treated with Apremilast on the in vitro wound healing of fibroblast and epithelial cell lines, we found a significant influence particularly on fibroblast wound closure. The presence of conditioned media of Apremilast-treated M1 macrophages significantly ameliorated wound closure in fibroblast cells. GO enrichment analysis showed that Apremilast treatment of M1 macrophages affects the expression of genes belonging to specific pathways that are linked to cell migration and cell division. In wound healing and fibrosis, both macrophages and fibroblasts are primary effector cells, and in tissue repair, one of the central roles played by macrophages is to activate fibroblasts. This is underlined by their inevitable interaction through the reciprocal exchange of growth factor signals and cytokines. Indeed, fibroblasts cultured with conditioned media from activated M1 and M2 macrophages portray phenotypic changes in fibroblasts [46,47]. Additionally, paracrine factors of M1 macrophages led to an increase in inflammatory cytokines and MMPs, and most interestingly, M2 macrophages influenced the proliferation rate of fibroblasts.

Macrophages are the main source of MMPs (MMP-1, -7, -8, -9, and -12), which have been shown to be crucial for inflammatory response and the progression of tissue remodeling [23,48]. MMP-9 was reported to possess the capacity to induce fibroblast migration [49]. We found MMP9 and MMP1 significantly upregulated in Apremilast-treated M1 macrophages, indicating their contribution to wound closure in the present study. In addition to MMPs, macrophages are a leading producer of TGF-β [50], which acts as an important signal in macrophage–fibroblast interactions [51]. The growth factor exists in three isoforms (TGF-β1, TGF-β2, and TGF-β3) with three consecutive genes (TGFB1, TGFB2, TGFB3), and contributes as a profibrotic agent to tissue remodeling and fibrosis [23], exerting numerous effects on wound healing by regulating cell proliferation and migration [52]. In the present study, treatment with Apremilast resulted in the upregulation of TGFB2 and TGFB3 in M1 macrophages, whereas the conditioned medium of Apremilast-treated M1 macrophages induced improved wound healing. Previous studies already examined the effect of TGFBs on the proliferation and migration of the fibroblasts of different origins, [53,54] but to the best of our knowledge, the paracrine influence of TGFBs on intestinal fibroblasts has not been studied so far. Based on the results obtained from Apremilast treatment, we suggest TGFB2 and TGFB3 gene products TGF-β2 and TGF-β3 to affect fibroblast proliferation and migration in a paracrine manner.

In contrast to the distinct effects of Apremilast-treated macrophages on fibroblasts, there was no significant effect on epithelial migration. However, a direct effect of Apremilast on IECs has already been tested in animal models of acute and chronic erosive colitis, demonstrating reduced epithelial damage and an enhanced epithelial barrier function in Apremilast-treated mice [25,55], indicating a distinct effect of Apremilast on IECs. Thus, the missing effect might be due the used experimental assay, which rather focuses on IEC migration and proliferation than on direct anti-inflammatory effects or barrier function. Thus, further analysis is needed to better focus on the effect of Apremilast on IEC in the sequence of intestinal wound healing to elucidate their interaction and underlying mechanisms.

As mentioned above, the present study was designed to evaluate the influence of Apremilast-induced changes on macrophage polarization and their impact on intestinal wound healing. Thereby, we focused on fibroblasts’ and IECs’ migration and proliferation, which solely represents a small part of the complex mechanism in intestinal wound healing. The 3D cell culture models or co-culture models would better mimic the interaction of cells that are relevant for intestinal wound healing. Thereby, not only fibroblast and IECs should be evaluated but also neutrophils and platelets, which are known to be involved in intestinal wound healing [22,56,57] and a potential target of Apremilast [58,59]. Besides cell differentiation and proliferation, angiogenesis plays a crucial part in intestinal wound healing [60]. Further study should evaluate the effect of Apremilast on angiogenesis, especially via the section of vascular endothelial growth factor (VEGF) by macrophages. Lastly, the herein obtained results of a macrophage phenotype switch must be evaluated with regard to the potential inflammatory transformation into colorectal cancer (CRC). Macrophages are known to not only play an important role in intestinal inflammation and injury repair, but also in the occurrence and development of CRC [8,10]. Thus, the favorable role of M2 macrophages for intestinal wound healing (anti-inflammatory and pro-healing) might be less beneficial when acting as tumor-associated macrophages (TAMs) by preventing immune responses against tumor cells and promoting the growth of these tumors [7]. This finding must be carefully evaluated when supporting the macrophage phenotype switch from M1 to M2 to ameliorate intestinal wound healing under inflammatory conditions.

## 5. Conclusions

In conclusion, we could ascertain a phenotype switch in macrophages from M1 to M2 through Apremilast under culture conditions and interference with NF-κB- and inflammatory pathways. Apremilast treatment positively influenced fibroblast migration and wound closure in vitro. Thus, our observation that the application of Apremilast is a promising target to ameliorate intestinal wound healing through its effect on macrophages raises important considerations for future therapeutic approaches.

## Data Availability

The datasets analyzed during the current study are available from the corresponding author on request.

## Supplementary Materials

The following supporting information can be downloaded at: https://www.mdpi.com/article/10.3390/jcm12103359/s1, Table S1: Complete list of differentially expressed genes (**A**) M1 and M2 polarization (**B**) GO:0006954 inflammatory response (**C**) GO:0043123 positive regulation of I-κB kinase/NF-κB signaling (**D**) GO:0042060 wound healing.

## References

[1] Turner J.R. (2009). Intestinal mucosal barrier function in health and disease. Nat. Rev. Immunol..

[2] Marjanovic G., Hopt U.T. (2011). Physiology of anastomotic healing. Chirurg.

[3] Quirós M., Nusrat A. (2019). Contribution of Wound-Associated Cells and Mediators in Orchestrating Gastrointestinal Mucosal Wound Repair. Annu. Rev. Physiol..

[4] Oishi Y., Manabe I. (2018). Macrophages in inflammation, repair and regeneration. Int. Immunol..

[5] Shi J., Wu Z., Li Z., Ji J. (2018). Roles of Macrophage Subtypes in Bowel Anastomotic Healing and Anastomotic Leakage. J. Immunol. Res..

[6] Na Y.R., Stakenborg M., Seok S.H., Matteoli G. (2019). Macrophages in intestinal inflammation and resolution: A potential therapeutic target in IBD. Nat. Rev. Gastroenterol. Hepatol..

[7] Isidro R.A., Appleyard C.B. (2016). Colonic macrophage polarization in homeostasis, inflammation, and cancer. Am. J. Physiol. Gastrointest. Liver Physiol..

[8] Lu L., Liu Y.J., Cheng P.Q., Hu D., Xu H.C., Ji G. (2021). Macrophages play a role in inflammatory transformation of colorectal cancer. World J. Gastrointest. Oncol..

[9] Kim S.Y., Nair M.G. (2019). Macrophages in wound healing: Activation and plasticity. Immunol. Cell Biol..

[10] Liu J., Geng X., Hou J., Wu G. (2021). New insights into M1/M2 macrophages: Key modulators in cancer progression. Cancer Cell Int..

[11] Ries C.H., Cannarile M.A., Hoves S., Benz J., Wartha K., Runza V., Rey-Giraud F., Pradel L.P., Feuerhake F., Klaman I. (2014). Targeting tumor-associated macrophages with anti-CSF-1R antibody reveals a strategy for cancer therapy. Cancer Cell.

[12] Ruffell B., Coussens L.M. (2015). Macrophages and therapeutic resistance in cancer. Cancer Cell.

[13] Afik R., Zigmond E., Vugman M., Klepfish M., Shimshoni E., Pasmanik-Chor M., Shenoy A., Bassat E., Halpern Z., Geiger T. (2016). Tumor macrophages are pivotal constructors of tumor collagenous matrix. J. Exp. Med..

[14] Tiainen S., Tumelius R., Rilla K., Hämäläinen K., Tammi M., Tammi R., Kosma V.M., Oikari S., Auvinen P. (2015). High numbers of macrophages, especially M2-like (CD163-positive), correlate with hyaluronan accumulation and poor outcome in breast cancer. Histopathology.

[15] Italiani P., Boraschi D. (2014). From Monocytes to M1/M2 Macrophages: Phenotypical vs. Functional Differentiation. Front. Immunol..

[16] Ferrante C.J., Leibovich S.J. (2012). Regulation of Macrophage Polarization and Wound Healing. Adv. Wound Care.

[17] Winter M., Heitplatz B., Koppers N., Mohr A., Bungert A.D., Juratli M.A., Strücker B., Varga G., Pascher A., Becker F. (2023). The Impact of Phase-Specific Macrophage Depletion on Intestinal Anastomotic Healing. Cells.

[18] Becker F., Kurmaeva E., Gavins F.N., Stevenson E.V., Navratil A.R., Jin L., Tsunoda I., Orr A.W., Alexander J.S., Ostanin D.V. (2016). A Critical Role for Monocytes/Macrophages During Intestinal Inflammation-associated Lymphangiogenesis. Inflamm. Bowel. Dis..

[19] Moreira Lopes T.C., Mosser D.M., Gonçalves R. (2020). Macrophage polarization in intestinal inflammation and gut homeostasis. Inflamm. Res..

[20] Martin K.E., García A.J. (2021). Macrophage phenotypes in tissue repair and the foreign body response: Implications for biomaterial-based regenerative medicine strategies. Acta Biomater..

[21] Sommer K., Wiendl M., Müller T.M., Heidbreder K., Voskens C., Neurath M.F., Zundler S. (2021). Intestinal Mucosal Wound Healing and Barrier Integrity in IBD-Crosstalk and Trafficking of Cellular Players. Front. Med..

[22] Xue X., Falcon D.M. (2019). The Role of Immune Cells and Cytokines in Intestinal Wound Healing. Int. J. Mol. Sci..

[23] Van Linthout S., Miteva K., Tschöpe C. (2014). Crosstalk between fibroblasts and inflammatory cells. Cardiovasc. Res..

[24] Ma S., Zhang J., Liu H., Li S., Wang Q. (2022). The Role of Tissue-Resident Macrophages in the Development and Treatment of Inflammatory Bowel Disease. Front. Cell Dev. Biol..

[25] Li H., Fan C., Feng C., Wu Y., Lu H., He P., Yang X., Zhu F., Qi Q., Gao Y. (2019). Inhibition of phosphodiesterase-4 attenuates murine ulcerative colitis through interference with mucosal immunity. Br. J. Pharmacol..

[26] Danese S., Neurath M.F., Kopoń A., Zakko S.F., Simmons T.C., Fogel R., Siegel C.A., Panaccione R., Zhan X., Usiskin K. (2020). Effects of Apremilast, an Oral Inhibitor of Phosphodiesterase 4, in a Randomized Trial of Patients With Active Ulcerative Colitis. Clin. Gastroenterol. Hepatol..

[27] Genin M., Clement F., Fattaccioli A., Raes M., Michiels C. (2015). M1 and M2 macrophages derived from THP-1 cells differentially modulate the response of cancer cells to etoposide. BMC Cancer.

[28] Bolger A.M., Lohse M., Usadel B. (2014). Trimmomatic: A flexible trimmer for Illumina sequence data. Bioinformatics.

[29] Kim D., Langmead B., Salzberg S.L. (2015). HISAT: A fast spliced aligner with low memory requirements. Nat. Methods.

[30] Li H. (2011). A statistical framework for SNP calling, mutation discovery, association mapping and population genetical parameter estimation from sequencing data. Bioinformatics.

[31] Anders S., Pyl P.T., Huber W. (2015). HTSeq—A Python framework to work with high-throughput sequencing data. Bioinformatics.

[32] Love M.I., Huber W., Anders S. (2014). Moderated estimation of fold change and dispersion for RNA-seq data with DESeq2. Genome Biol..

[33] Juhas U., Ryba-Stanisławowska M., Szargiej P., Myśliwska J. (2015). Different pathways of macrophage activation and polarization. Postepy. Hig. Med. Dosw..

[34] Lawrence T., Natoli G. (2011). Transcriptional regulation of macrophage polarization: Enabling diversity with identity. Nat. Rev. Immunol..

[35] Rőszer T. (2015). Understanding the Mysterious M2 Macrophage through Activation Markers and Effector Mechanisms. Mediators Inflamm..

[36] https://david.ncifcrf.gov/home.jsp.

[37] Lu Q.K., Fan C., Xiang C.G., Wu B., Lu H.M., Feng C.L., Yang X.Q., Li H., Tang W. (2022). Inhibition of PDE4 by apremilast attenuates skin fibrosis through directly suppressing activation of M1 and T cells. Acta Pharmacol. Sin..

[38] Schafer P.H., Parton A., Capone L., Cedzik D., Brady H., Evans J.F., Man H.W., Muller G.W., Stirling D.I., Chopra R. (2014). Apremilast is a selective PDE4 inhibitor with regulatory effects on innate immunity. Cell Signal.

[39] Abdulrahim H., Thistleton S., Adebajo A.O., Shaw T., Edwards C., Wells A. (2015). Apremilast: A PDE4 inhibitor for the treatment of psoriatic arthritis. Expert Opin. Pharmacother..

[40] Paul C., Cather J., Gooderham M., Poulin Y., Mrowietz U., Ferrandiz C., Crowley J., Hu C., Stevens R.M., Shah K. (2015). Efficacy and safety of apremilast, an oral phosphodiesterase 4 inhibitor, in patients with moderate-to-severe plaque psoriasis over 52 weeks: A phase III, randomized controlled trial (ESTEEM 2). Br. J. Dermatol..

[41] Nassim D., Alajmi A., Jfri A., Pehr K. (2020). Apremilast in dermatology: A review of literature. Dermatol. Ther..

[42] Schafer P.H., Parton A., Gandhi A.K., Capone L., Adams M., Wu L., Bartlett J.B., Loveland M.A., Gilhar A., Cheung Y.F. (2010). Apremilast, a cAMP phosphodiesterase-4 inhibitor, demonstrates anti-inflammatory activity in vitro and in a model of psoriasis. Br. J. Pharmacol..

[43] Maloney N.J., Zhao J., Tegtmeyer K., Lee E.Y., Cheng K. (2020). Off-label studies on apremilast in dermatology: A review. J. Dermatolog. Treat..

[44] Schafer P.H., Truzzi F., Parton A., Wu L., Kosek J., Zhang L.H., Horan G., Saltari A., Quadri M., Lotti R. (2016). Phosphodiesterase 4 in inflammatory diseases: Effects of apremilast in psoriatic blood and in dermal myofibroblasts through the PDE4/CD271 complex. Cell Signal.

[45] Wu X., Wang Z., Shi J., Yu X., Li C., Liu J., Zhang F., Chen H., Zheng W. (2022). Macrophage polarization toward M1 phenotype through NF-κB signaling in patients with Behçet’s disease. Arthritis. Res. Ther..

[46] Ploeger D.T., Hosper N.A., Schipper M., Koerts J.A., de Rond S., Bank R.A. (2013). Cell plasticity in wound healing: Paracrine factors of M1/M2 polarized macrophages influence the phenotypical state of dermal fibroblasts. Cell Commun. Signal..

[47] Li Z., Bratlie K.M. (2021). Fibroblasts treated with macrophage conditioned medium results in phenotypic shifts and changes in collagen organization. Mater. Sci. Eng. C Mater. Biol. Appl..

[48] Murray P.J., Wynn T.A. (2011). Protective and pathogenic functions of macrophage subsets. Nat. Rev. Immunol..

[49] Robert S., Gicquel T., Victoni T., Valença S., Barreto E., Bailly-Maître B., Boichot E., Lagente V. (2016). Involvement of matrix metalloproteinases (MMPs) and inflammasome pathway in molecular mechanisms of fibrosis. Biosci. Rep..

[50] Verrecchia F., Mauviel A. (2007). Transforming growth factor-beta and fibrosis. World J. Gastroenterol..

[51] Buechler M.B., Fu W., Turley S.J. (2021). Fibroblast-macrophage reciprocal interactions in health, fibrosis, and cancer. Immunity.

[52] Lichtman M.K., Otero-Vinas M., Falanga V. (2016). Transforming growth factor beta (TGF-β) isoforms in wound healing and fibrosis. Wound Repair Regen..

[53] Cordeiro M.F., Bhattacharya S.S., Schultz G.S., Khaw P.T. (2000). TGF-beta1, -beta2, and -beta3 in vitro: Biphasic effects on Tenon’s fibroblast contraction, proliferation, and migration. Investig. Ophthalmol. Vis. Sci..

[54] Ueshima E., Fujimori M., Kodama H., Felsen D., Chen J., Durack J.C., Solomon S.B., Coleman J.A., Srimathveeravalli G. (2019). Macrophage-secreted TGF-β(1) contributes to fibroblast activation and ureteral stricture after ablation injury. Am. J. Physiol. Renal. Physiol..

[55] Li H., Zhang Y., Liu M., Fan C., Feng C., Lu Q., Xiang C., Lu H., Yang X., Wu B. (2022). Targeting PDE4 as a promising therapeutic strategy in chronic ulcerative colitis through modulating mucosal homeostasis. Acta Pharm. Sin. B.

[56] Fresno L., Fondevila D., Bambo O., Chacaltana A., García F., Andaluz A. (2010). Effects of platelet-rich plasma on intestinal wound healing in pigs. Vet. J..

[57] Zhang F., Qiao S., Li C., Wu B., Reischl S., Neumann P.A. (2020). The immunologic changes during different phases of intestinal anastomotic healing. J. Clin. Lab. Anal..

[58] Schett G., Sloan V.S., Stevens R.M., Schafer P. (2010). Apremilast: A novel PDE4 inhibitor in the treatment of autoimmune and inflammatory diseases. Ther. Adv. Musculoskelet. Dis..

[59] Tsai Y.F., Chen C.Y., Yang S.C., Syu Y.T., Hwang T.L. (2022). Apremilast ameliorates acute respiratory distress syndrome by inhibiting neutrophil-induced oxidative stress. Biomed. J..

[60] Villablanca E.J., Selin K., Hedin C.R.H. (2022). Mechanisms of mucosal healing: Treating inflammatory bowel disease without immunosuppression?. Nat. Rev. Gastroenterol. Hepatol..

